# Outcomes Following Close Collaboration With Parents Intervention in Neonatal Intensive Care Units: A Nonrandomized Clinical Trial

**DOI:** 10.1001/jamanetworkopen.2024.54099

**Published:** 2025-01-09

**Authors:** Ryo Itoshima, Heili Varendi, Liis Toome, Pille Saik, Anna Axelin, Liisa Lehtonen, Ali Moazami-Goodarzi, Sari Ahlqvist-Björkroth

**Affiliations:** 1 Department of Clinical Medicine, University of Turku, Turku, Finland; 2 Department of Paediatrics and Adolescent Medicine, Turku University Hospital, Turku, Finland; 3 Life Science Research Center, Nagano Children’s Hospital, Azumino, Japan; 4 Neonatal Unit, Children’s Clinic of Tartu University Hospital, Tartu, Estonia; 5 Department of Neonatal and Infant Medicine, Tallinn Children’s Hospital, Tallinn, Estonia; 6 Department of Neonatology, West-Tallinn Central Hospital, Tallinn, Estonia; 7 Department of Nursing Science, University of Turku, Turku, Finland; 8 Department of Psychology and Speech-Language Pathology, University of Turku, Turku, Finland

## Abstract

**Importance:**

Family-centered care (FCC) in neonatal intensive care units (NICUs) is critical for parental involvement and infant well-being, yet few studies have evaluated the impact of FCC interventions on practice or examined how implementation fidelity may affect these outcomes.

**Objectives:**

To evaluate the association between the Close Collaboration With Parents intervention and FCC practices and how implementation fidelity may modify these outcomes.

**Design, Setting, and Participants:**

This nonrandomized clinical trial had a before-and-after design. It was conducted between March 2021 and June 2023 at 6 NICUs in Estonia and included the parents of all newborns and all staff.

**Intervention:**

Close Collaboration With Parents is an educational intervention for multiprofessional NICU staff including e-learning and bedside practices combined with reflection sessions. It aims to improve FCC culture by developing staff skills in communication and collaboration with parents and parenting support.

**Main Outcomes and Measures:**

The quality of FCC was assessed using a 1-time questionnaire for the parents with 9 questions from the Digi Family-Centered Care–Parent version tool, and daily questions for the staff using Digi Family-Centered Care–Nurse version tool. The association between implementation fidelity, defined as the proportion of staff who completed the full education, and the quality of FCC was also analyzed.

**Results:**

A total of 186 infants (with responses from 186 mothers and 22 fathers) were included in the preintervention period. The median (IQR) gestational age was 37.4 (34.0-39.9) weeks, 94 infants (50.5%) were male, and the median (IQR) parental age was 32 (28-36) years. A total of 208 infants (with responses from 208 mothers and 55 fathers) were included in the postintervention period. The median (IQR) gestational age was 38.1 (35.4-39.9) weeks, 114 infants (55.1%) were male, and median (IQR) parental age was 32 (27-35) years. From the staff, 7448 and 6717 daily responses were analyzed in the preintervention and postintervention periods, respectively. The total rating was significantly higher after the intervention than before, by both the parents (*r* = 0.07; *P* < .001) and the staff (*r* = 0.10; *P* < .001). The median ratings did not, however, change: the parents’ median (IQR) overall rating before and after was 7.0 (6.0-7.0), and the staff’s median (IQR) overall rating was 6.0 (5.0-7.0) before and 6.0 (6.0-7.0) after. Implementation fidelity ranged from 4 of 30 health care professionals (13.3%) to 37 of 45 (82.2%) at the NICUs. The NICUs with high fidelity, as opposed to low fidelity, had significantly greater improvement in FCC ratings by the staff after the intervention (β = 2.1 [95% CI, 0.8-3.4]; *P* = .002).

**Conclusions and Relevance:**

In this nonrandomized clinical trial, FCC practices were rated higher by the staff and parents after the Close Collaboration With Parents intervention. Implementation fidelity was identified as an important factor.

**Trial Registration:**

ClinicalTrials.gov Identifier: NCT06258655

## Key Points

**Question:**

Is the Close Collaboration With Parents, an educational intervention for health care staff, associated with improved ratings from parents and staff on the quality of family-centered care in neonatal intensive care units, and does implementation fidelity modify this association?

**Findings:**

In this nonrandomized clinical trial conducted at 6 neonatal intensive care units with 393 infants, the intervention was associated with improvements in family-centered care practices as rated by both the parents and health care staff. Implementation fidelity was a significant modifier of the ratings by the health care staff.

**Meaning:**

These findings suggest that family-centered care practices can be modified by educational interventions, but attention should be paid to implementation fidelity to achieve the desired changes.

## Teaser

This nonrandomized clinical trial evaluates whether an educational program for neonatal intensive care unit (NICU) staff on family-centered care is associated with parent and staff ratings of family-centered care practices and whether fidelity to the program modifies this association.

## Introduction

Family-centered care (FCC) in neonatal intensive care units (NICUs) is an approach to provide neonatal care in partnership with the newborn’s family.^1,2^ Core principles include respect and dignity, information sharing, negotiation, participation, and collaboration, with mutual trust and shared responsibility.^3^ The health and parent organizations emphasize the rights of the parents and the importance of their role in newborn care and in decision-making as primary caregivers.^4,5,6^ Strong evidence also supports FCC as an essential part of modern neonatal care, bringing health benefits for both parents and infants.^7,8^

Although FCC is widely advocated, its implementation in everyday care remains challenging. The implementation requires changes in the mindsets and behaviors of staff as well as in the organizational structure of the NICU.^3,9,10^ Although the effects of FCC interventions have been studied on several outcomes, limited knowledge exists on their impact on FCC practices. There is also a gap in the knowledge of the implementation of these interventions, ie, whether they happen as intended, also called fidelity.^11^

The Close Collaboration With Parents intervention is a structured educational program for the NICU staff to improve FCC. The intervention improved FCC practices in 8 Finnish NICUs, with implementation fidelity ranging from 46% to 100%.^12,13,14^ The observed variation in fidelity provided a reason to study its role further. In addition, it is of interest whether the intervention can change FCC practices internationally. In this study, we aimed to evaluate the effectiveness and fidelity of the Close Collaboration With Parents intervention and the role of fidelity in the improvement of FCC practices in 6 NICUs in Estonia.

## Methods

### Study Design and Population

This prospective nonrandomized clinical trial used before-and-after comparisons. There are 7 NICUs in Estonia: 1 level IV unit in Tartu, 1 level III unit in Tallinn, and 5 level II units. The level IV and III units are combined with pediatric intensive care units. Almost all infants treated in level III or IV units are transferred to level II units, which function as step-down or intermediate units.

The study sites consisted of 6 NICUs in 3 hospitals in Estonia. The Close Collaboration With Parents intervention was implemented at all study sites between September 2021 and December 2022. The data were collected between March and August 2021 for the preintervention period and between December 2022 and June 2023 for the postintervention period (eFigure in Supplement 1). We aimed to recruit 50 families in each hospital before and after the intervention based on the power calculations to detect an association between the intervention and parental depression.^15,16^

The study population consisted of parents and NICU staff. Parents were eligible if (1) the newborn infant was admitted to the NICUs before 28 days of age, (2) the expected length of stay was at least 3 days, and (3) they could understand Estonian, Russian, or English. All staff who worked during the study periods were eligible, including neonatologists, pediatricians, anesthesiologists, registered nurses, assistant nurses, and any other staff.

This study followed the Transparent Reporting of Evaluations with Nonrandomized Designs (TREND) reporting guideline.^17^ Ethical approval was received from the Research Ethics Committee of the University of Tartu. Written informed consent was obtained from all participants. The study protocol appears in Supplement 2.

### Intervention

Close Collaboration With Parents is an educational intervention for multiprofessional NICU staff.^12,18,19,20^ It consists of 4 training phases with the following objectives: (1) learning systematic observation of infant behavior, (2) performing joint infant observations to understand the infant’s preferences and to plan infant care with the parents, (3) understanding the family’s individual story using a semistructured discussion, and (4) including the parents in decision-making during medical rounds and discharge preparation.^20^ The learning process includes completing the e-learning module and bedside practices combined with reflection on the practice experience with a local mentor. The goal is to build staff communication skills to improve collaboration with and support for parents.

The train-the-trainer model was used in the implementation. The training team trained local mentors in each NICU, who then trained the other staff in their unit using their native language.^20^ In Estonia, most training sessions for local mentors were conducted remotely because of the COVID-19 pandemic. When the local mentors trained the staff, the training team offered 4 or 5 support visits per NICU to ensure the quality of the implementation and to help the local mentors solve implementation problems.

### Outcome Measures

Implementation fidelity has 5 components: adherence to the intervention protocol, dose and amount of intervention delivery, quality of delivery, participant responsiveness, and program differentiation based on core elements of intervention.^11,21^ We calculated the fidelity rate to measure the dose and amount of the intervention delivery, which was defined as the proportion of physicians and registered nurses who completed the full training. Fidelity rate was calculated using the total number of physicians and registered nurses at each NICU at the beginning of the study as the denominator, to account for staff turnover across the study period. The e-learning module automatically recorded each staff member’s use of the tool. Bedside training and reflection sessions were recorded in a training log when they happened.

The parents’ experiences of FCC were assessed using a questionnaire based on the Digi Family-Centered Care–Patient version.^22^ It consisted of 9 questions: (1) active listening, (2) parent participation in infant care, (3) individualized parent guidance, (4) shared decision-making, (5) the parent’s trust in staff, (6) the staff’s trust in parents, (7) individual information sharing, (8) emotional support, and (9) participation in medical rounds. Each question had response categories from 1 to 7, and a higher score indicated better FCC provision. A response of 0 was provided for cases where the parent did not visit the NICU or participate in the medical rounds. Parents answered the questionnaire when their infant was transferred to another NICU and discharged home, the earliest 4 days before and the latest on the same day of the transfer or discharge. The Cronbach α of the responses from the parents was 0.85 in the preintervention group and 0.91 in the postintervention group.

The staff members’ daily experiences of their FCC practices were assessed using the Digi Family-Centered Care–Nurse version tool.^22^ It was a web-based questionnaire with 9 questions, which were the same as those used for the parents but presented from the staff’s perspective. A response of 0 was provided if the staff did not work with parents or participate in the medical rounds. The staff answered 3 randomly selected questions (of 9) on a computer after each working shift. One or more computers dedicated to research use were placed in each NICU.

Our primary outcomes were the parents’ and staff’s experiences of the level of FCC practices in their NICU. Furthermore, we evaluated the fidelity rate achieved by each NICU and its association with the level of FCC practices.

### Statistical Analysis

The Wilcoxon rank sum test was used to compare parents’ ratings of FCC experiences before and after the intervention. Because most responses clustered toward the higher end of the scale (5-7), this nonparametric test was chosen for its robustness and greater power than the parametric *t* test with skewed data. The Wilcoxon test better maintains type I error rates at the nominal level and reduces the risk of type III errors, ensuring more reliable conclusions.^23,24^

The linear regression model was used to adjust for multiple variables, including infants’ birth weight, parental native language (categorized as Estonian or Russian vs others), NICU level (level II vs III or IV) and the NICU fidelity rate (classified as low vs high, divided by the median). We included birth weight because it differed between the groups. Gestational age and length of stay were not included because they were correlated with birth weight. The inclusion of native language in the model was justified by its potential influence on staff-parent communication, which may subsequently affect Family-Centered Care (FCC) practices. Estonian and Russian are the 2 predominant languages spoken in Estonia, making this adjustment relevant. Additionally, the fidelity rate and the NICU level were adjusted for when analyzing staff-related outcomes. To approximate a normal distribution for the model residuals, Box-Cox transformation was applied to the dependent variables, and model fit was evaluated using the Shapiro-Wilk *W* statistic. Separate linear regression models were used to assess the association between fidelity rate and changes in the ratings of all questionnaire items before and after the intervention. When the parent did not visit the NICU or participate in medical rounds, a response of 0 was recorded. The dataset contained minimal missing values, with less than 1.74% in the parent sample and 1.54% in the staff sample. A missing data pattern analysis confirmed that the missing data were completely at random. Consequently, listwise deletion was applied to the analyses of missing data. All statistical analyses were performed using R version 4.2.2 (R Project for Statistical Computing) with the Tidyverse (version 1.3.2),^25^ AID (version 2.9),^26^ and lme4 (version 1.1-31).^27^ Visualization was conducted using the ggplot2 (version 3.4.0).^28^ A threshold of *P* < .05 was set for statistical significance, and all tests were 2-tailed.

## Results

Of the 156 NICU staff members, 99 completed the training, including 21 physicians (21%) and 57 nurses or midwives (58%). Half of physicians and nurses completed the training from phase 1 to 4. The fidelity rates were higher than the median in 3 NICUs (37 of 45 [82.2%] in NICU E and F and 13 of 18 [72.2%] in NICU C) and lower in the other 3 NICUs (16 of 34 [47.1%] in NICU B, 8 of 29 [27.6%] in NICU A, and 4 of 30 [13.3%] in NICU D) (Table 1).

**Table 1.** Status and Fidelity of the Implementation in Each Unit

NICU	Level of care	No. of physicians and RNs^c^	Completed staff^a^							Completed physicians and RNs, No. (%)^b^			
			Total		Physician	RN^d^		Other		Until phase 1	Until phase 2	Until phase 3	Until phase 4 (fidelity rate)^e^
A	III	29	8	0			8		0	20 (69.0)	16 (55.2)	13 (44.8)	8 (27.6)
B	II	34	29	3			13		13	27 (79.4)	23 (67.6)	21 (61.8)	16 (47.1)
C	II	18	14	6			7		1	20 (111.1)^f^	18 (100.0)	16 (88.9)	13 (72.2)
D	IV	30	4	1			3		0	22 (73.3)	19 (63.3)	6 (20.0)	4 (13.3)
E and F^g^	II	45	44	11			26		7	48 (106.7)^f^	44 (97.8)	41 (91.1)	37 (82.2)
Total	NA	156	99	21			57		21	137 (87.8)	120 (76.9)	97 (62.2)	78 (50.0)

Abbreviations: NA, not applicable; NICU, neonatal intensive care unit; RN registered nurse.

^a^ Completing the full training, including e-learning, bedside training, and reflections, in all 4 training phases.

^b^ Completion of each training phase was defined as completing the e-learning module and participating in at least 1 bedside training and reflection session.

^c^ Physicians and registered nurses at the beginning of the preintervention data collection.

^d^ Including midwives.

^e^ The fidelity rate of the implementation of the intervention was defined as the proportion of physicians and RNs who completed all training phases (until phase 4).

^f^ The total number of physicians and RNs who had completed the training was more than the number of them at the beginning of the study.

^g^ NICU E and F were merged in the fidelity calculation because there were occasional staff sharing and movements between these units.

**Figure 1.**
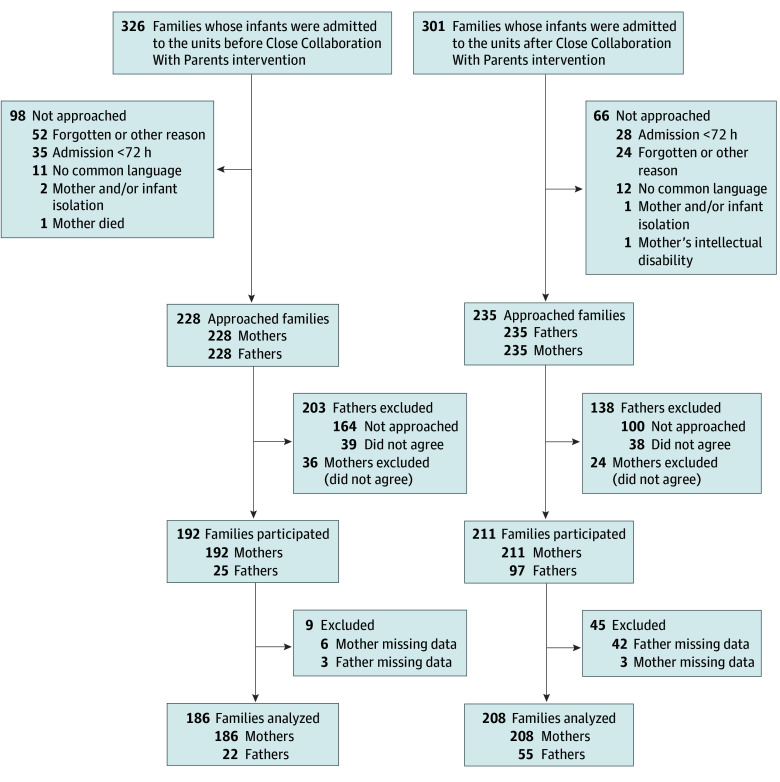
Flow Diagram of Family Recruitment and Participation

Of 228 families approached before the intervention and 235 after the intervention, 192 mothers and 25 fathers agreed to participate before the intervention, and 211 mothers and 97 fathers agreed after the intervention. Responses from 186 mothers and 22 fathers before the intervention (median [IQR] parental age, 32 [28-36] years; 94 [50.5%] male infants) and 208 mothers and 55 fathers after the intervention were included in the analyses (median [IQR] parental age, 32 [27-35] years; 114 [55.1%] male infants) (Figure 1). Among the analyzed families, 44 families before the intervention and 28 after experienced a transfer of their infants to another study site. The postintervention group included infants who were born at higher gestational age than the preintervention group (median [IQR], 38.1 [35.4-39.9] vs 37.4 [34.0-39.9] weeks) and born at higher birth weights (median [IQR], 3275 [2482-3778] vs 2876 [2014-3618] g); fewer infants in the postintervention group than the preintervention group were admitted to level IV or III NICUs (26 [12.6%] vs 56 [30.1%]). The other characteristics were comparable between the 2 groups (Table 2).

**Table 2.** Characteristics of Infants and Parents in the Final Study Groups

Characteristic	Participants, No. (%)	
	Before intervention	After intervention
**Infant**		
No.	186	208
Gestational age, median (IQR), wk	37.4 (34.0-39.9)	38.1 (35.4-39.9)
Birth weight, median (IQR), g	2876 (2014-3618)	3275 (2482-3778)
Length of hospital stay, median (IQR), d	9.5 (6.0-25.5)	9.0 (6.0-19.0)
Sex		
Male	94 (50.5)	114 (55.1)
Female	92 (49.5)	93 (44.9)
Cesarean delivery	77 (41.4)	81 (39.1)
Singleton	166 (89.2)	199 (96.1)
Admission to level III NICU	56 (30.1)	26 (12.6)
**Parents**		
No.		
Mothers	186	208
Fathers	22	55
Age, median (IQR), y	32 (28-36)	32 (27-35)
Single parent	1 (0.5)	6 (2.3)
No siblings at home	96 (48.2)	137 (55.5)
Higher education^a^	101 (52.3)	141 (57.3)
Native Estonian or Russian speaker	203 (98.1)	244 (94.9)
In paid work	158 (77.8)	194 (75.8)
Smoker	12 (5.8)	22 (8.6)
Previous depression or anxiety	16 (7.7)	23 (8.9)

Abbreviation: NICU, neonatal intensive care unit.

^a^ Bachelor’s degree or higher.

**Figure 2.**
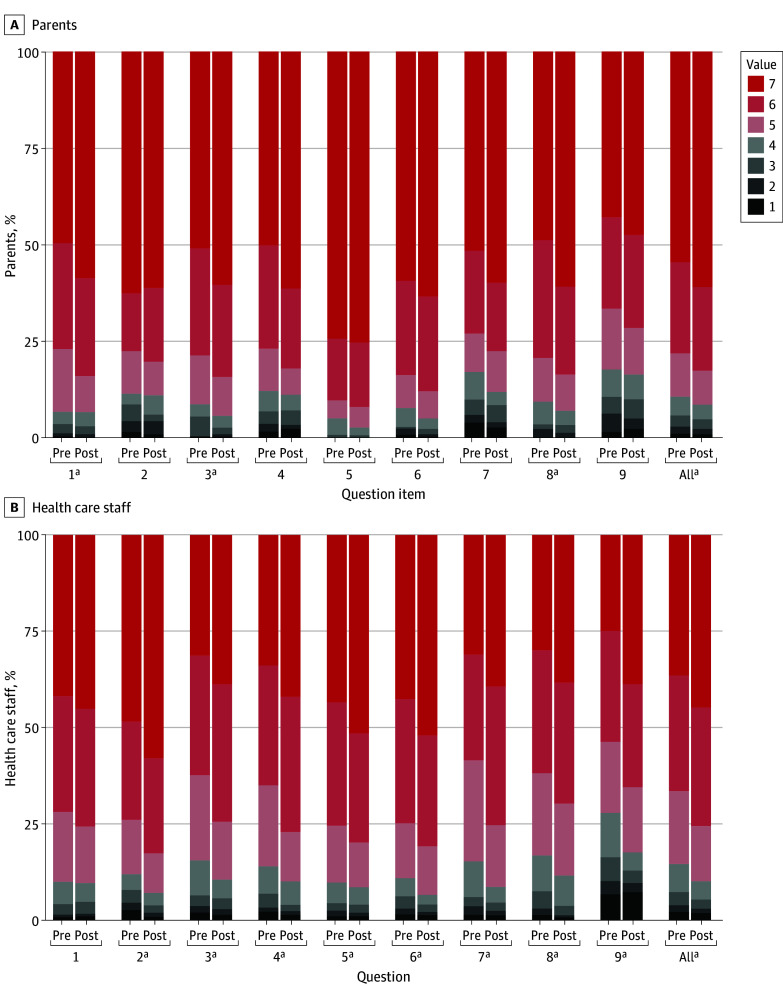
Changes in the Ratings of Family-Centered Care by Parents and Health Care Staff Before and After Intervention Questions appear in Table 3 and the Methods section. ^a^A statistically significant change after intervention based on the results of the linear regression models.

The total ratings including all responses from parents increased significantly based on the change in the distribution of the ratings (*r* = 0.07; *P* < .001) (Figure 2). However, the overall median (IQR) did not change, likely due to the high baseline level (before, 7.0 [6.0-7.0]; after, 7.0 [6.0-7.0]). The ratings improved significantly after the intervention in the following questions: active listening, individualized parent guidance, shared decision-making, and emotional support. The same items remained significant in the linear regression models, except for shared decision-making (Table 3).

**Table 3.** Comparison of Parent and Staff Ratings of Family-Centered Care Practices Before and After the Intervention

Component	No. of responses (No. missing)		Median (IQR)		After vs before^a^		Linear regression model^b^	
	Before	After	Before	After	*r*	*P* value	β (95% CI)	*P* value
**Parents**								
Q1, active listening	252 (6)	300 (1)	6.0 (6.0 to 7.0)	7.0 (6.0 to 7.0)	.10	.03	6.3 (0.62 to 12.0)	.03
Q2, participation in care	254 (4)	299 (2)	7.0 (6.0 to 7.0)	7.0 (6.0 to 7.0)	.00	.98	−2.0 (−13.0 to 8.9)	.70
Q3, individualized guidance	253 (5)	298 (3)	7.0 (6.0 to 7.0)	7.0 (6.0 to 7.0)	.10	.02	9.5 (1.4 to 17.0)	.02
Q4, shared decision-making	247 (11)	295 (6)	7.0 (6.0 to 7.0)	7.0 (6.0 to 7.0)	.10	.02	13 (−0.07 to 26.0)	.05
Q5, parent trust in staff	257 (1)	300 (1)	7.0 (6.0 to 7.0)	7.0 (7.0 to 7.0)	.02	.69	9.6 (−45.0 to 65.0)	.70
Q6, staff trust in parent	246 (12)	298 (3)	7.0 (6.0 to 7.0)	7.0 (6.0 to 7.0)	.05	.23	10.0 (−6.1 to 26.0)	.20
Q7, individualized information	252 (6)	294 (7)	7.0 (5.0 to 7.0)	7.0 (6.0 to 7.0)	.08	.05	6.4 (−0.91 to 14.0)	.09
Q8, emotional support	256 (2)	299 (2)	6.0 (6.0 to 7.0)	7.0 (6.0 to 7.0)	.11	.007	10.0 (1.8 to 19.0)	.02
Q9, participation in medical rounds	254 (4)	299 (2)	6.0 (5.0 to 7.0)	6.0 (5.0 to 7.0)	.05	.24	1.6 (−1.9 to 5.0)	.40
All questions	2271 (51)	2682 (27)	7.0 (6.0 to 7.0)	7.0 (6.0 to 7.0)	.07	<.001	7.5 (4.0 to 11.0)	<.001
**Staff**								
Q1, active listening	849 (0)	743 (0)	6.0 (5.0 to 7.0)	6.0 (6.0 to 7.0)	.04	.12	0.3 (−1.3 to 2.0)	.70
Q2, participation in care	843 (0)	741 (0)	6.0 (5.0 to 7.0)	7.0 (6.0 to 7.0)	.11	<.001	8.2 (3.7 to 13.0)	<.001
Q3, individualized guidance	828 (0)	746 (0)	6.0 (5.0 to 7.0)	6.0 (5.0 to 7.0)	.12	<.001	2.6 (1.1 to 4.0)	<.001
Q4, shared decision-making	833 (0)	741 (0)	6.0 (5.0 to 7.0)	6.0 (6.0 to 7.0)	.12	<.001	3.4 (1.7 to 5.2)	<.001
Q5, parent trust in staff	822 (0)	732 (0)	6.0 (6.0 to 7.0)	7.0 (6.0 to 7.0)	.08	.002	3.5 (0.6 to 6.5)	.02
Q6, staff trust in parent	829 (0)	723 (0)	6.0 (5.0 to 7.0)	7.0 (6.0 to 7.0)	.10	<.001	5.4 (1.9 to 9.0)	.003
Q7, individualized information	809 (0)	748 (0)	6.0 (5.0 to 7.0)	6.0 (6.0 to 7.0)	.15	<.001	3.1 (1.9 to 4.2)	<.001
Q8, emotional support	843 (0)	739 (0)	6.0 (5.0 to 7.0)	6.0 (5.0 to 7.0)	.10	<.001	1.6 (0.7 to 2.5)	<.001
Q9, participation in medical rounds	792 (0)	804 (0)	6.0 (4.0 to 6.0)	6.0 (5.0 to 7.0)	.15	<.001	3.8 (2.3 to 5.3)	<.001
All questions	7448 (0)	6717 (0)	6.0 (5.0 to 7.0)	6.0 (6.0 to 7.0)	.10	<.001	3.3 (2.6 to 3.9)	<.001

Abbreviations: NICU, neonatal intensive care unit; Q, question.

^a^ Using Wilcoxon rank sum test; *r *is the effect size.

^b^ Box-Cox transformation was adopted for the dependent variables and β was calculated with the transformed variables. The model for parents adjusted for infants’ birth weight, if the parents’ native languages were either Estonian or Russian, the care level (level II vs III or IV) and the fidelity rate of the NICU (high vs low). The model for staff adjusted for the care level (level II vs III or IV) and the fidelity rate of the NICU (high vs low).

The staff gave 7448 responses before and 6717 after the intervention (eTable 1 in Supplement 1). Among the staff, the proportion of the highest rating (7) increased in all questions after the intervention (Figure 2). The total ratings including all responses from the staff increased significantly, (*r* = 0.10; *P* < .001). The overall staff median (IQR) did not change due to the high baseline (before, 6.0 [5.0-7.0]; after, 6.0 [6.0-7.0]) (Table 3). After the intervention, the median values improved significantly in all other of the 9 questions except for active listening.

The NICUs with a high fidelity rate, as opposed to low, had significantly greater improvement in FCC ratings by the staff after intervention in the linear regression model (β = 2.1 [95% CI, 0.8 to 3.4]; *P* = .002). On the other hand, the fidelity rate was not associated with the change in the FCC ratings by parents after the intervention (β = 2.7 [95% CI, −4.4 to 9.8]; *P* = .46) (eTable 2 in Supplement 1).

## Discussion

Our study showed that both the parents and staff reported better FCC practices after the Close Collaboration With Parents intervention. The staff reported a wider range of improvements than parents. We found a large variation in the implementation fidelity between the NICUs. Importantly, high implementation fidelity was associated with greater improvement in FCC practices as rated by staff.

In our study, active listening, individual guidance, and emotional support rated by the parents improved significantly, contrary to the previous studies using the same evaluation method.^13^ The parents’ experience of being listened to can be a prerequisite for emotional support and for building and maintaining a good staff-parent relationship.^29^ The Close Collaboration With Parents intervention explicitly provides training to the staff about the importance of active listening and individual guidance and how to facilitate them at the communicational level.^20^ On the other hand, shared decision-making improved only in the previous study.^13^ The different results in our study and the previous study using the same evaluation method can be explained by different baseline ratings. In addition, both shared decision-making and emotional support have been shown to be components of FCC that are difficult to improve.^13,30^

More items rated by the staff improved in our study than in the previous study.^13^ Both reported improved emotional support and the parent’s trust in staff as well as the total rating. The Close Collaboration With Parents intervention provides tools for the staff to learn about emotional support based on their individual needs. Empathetic communication impacts parents’ coping, knowledge, participation, and satisfaction and possibly reduces their stress.^31^ However, active listening as rated by the staff, which improved in the previous study, did not improve in this study. The other FCC items improved only in this study. The differences in the sample size and the analytic methods may have affected the different results. In addition, more physicians completed the implementation in our study (22% of all staff) than in the previous study (6%). The physicians’ involvement is a meaningful factor in practicing FCC.^12,32^

Taken together, we found improvements in the FCC practices in 6 Estonian NICUs. Many of these improvements were observed in staff-parent communication items, similar to the findings from studies examining the intervention in Finnish NICUs.^12,13,14^ Staff-parent communication is one of the key elements of FCC.^31^ Improving staff-parent communication by educating the NICU staff is included in several interventions.^33,34^ However, the other interventions have not been evaluated for their association with communication outcomes. A systematic review showed that effective staff-parent communication can reduce parents’ stress, improve their participation in infant care and communication about it, and improve their bonding with the infant.^31^ Therefore, future studies of FCC interventions should consider examining FCC practices and staff-parent communication as important proximal outcomes and potential mediators of short- and long-term outcomes.

The parent-staff difference in the intervention’s impact could be explained by the baseline ratings as parents’ baseline ratings were close to the maximum. In addition, a longer time could have been needed for the staff to change their behavior toward the parents. In the process of health care behavior change, a change in mindset happens first, followed by a change in behavior.^35^

To our knowledge, this is the first study to evaluate the association between implementation fidelity of an FCC intervention and FCC practices. Understanding the factors contributing to low fidelity is important for successful implementation in the future. One of the main factors could be the physicians’ low contribution to the process, which is one of the key factors of successful FCC.^12^ The 3 NICUs with low fidelity may have had difficulty in involving physicians in the project, as they did not have any physicians in the mentoring team. Meanwhile, even among the NICUs with low fidelity, the completion rate until Phase 2 training was greater than 55%. The implementation period may not have been long enough to complete the full training in the low-fidelity NICUs, as they had more staff to be trained than the other NICUs. In addition, these level III and IV NICUs consisted of open-bay rooms, while the others consisted of single-family rooms. The daily presence of parents in NICUs has been shown to be shorter in open-bay rooms than in single-family rooms,^36^ which may have made it more difficult for the mentors to find a parent for bedside training sessions.

Including the perspectives of both parents and staff was a strength of our study. We also included the families of a full range of neonatal patient populations, from preterm to full-term infants with various complications. Furthermore, we included all professionals working in the NICUs, as they all contribute to care culture of the unit.

### Limitations

This study has limitations. First, the effect size was small in most comparisons—even if statistically significant—because of the ceiling effect. The self-assessment tools might not be optimally sensitive to evaluate FCC.^37^ There is a need to develop more appropriate tools to evaluate FCC practices or to conduct ethnographic observations to validate their changes. Second, questions were presented differently to parents and staff, as prior research suggests daily answers from parents may not be more sensitive than 1-point measurement at discharge.^38^ Third, there was also an imbalance in the number of mothers and fathers due to the fathers’ limited access during the COVID-19 pandemic.^39^ Fourth, the staff responses were provided by all the staff, yet the calculation of the fidelity rate excluded temporary staff, assistant nurses, and special workers, who were not expected to receive the full training. Fifth, potential response bias in the staff responses was present in NICU A and D, where the staff responses decreased by about half after the intervention. The anonymous responses prevented further analysis. Furthermore, the quantitative evaluation of fidelity may be prone to random variation, especially in small units. Additionally, the intervention delivery and participant responsiveness should also be evaluated qualitatively in future studies.

## Conclusions

Our study aligns with previous findings that FCC practices in NICUs improved after the implementation of the Close Collaboration With Parents intervention. In addition, our study found that high fidelity was important in achieving these improvements. In the NICUs in Estonia, staff reported greater improvement in FCC practices than parents after the intervention. The intervention seemed to particularly improve the parents’ experience of shared decision-making, the staff’s ability to provide emotional support to the parents, and the parents’ trust in the staff. Future research should focus more on understanding how to achieve high fidelity for the greatest intervention impact.

## Notes

Supplement 1. **eFigure.** Periods of Intervention and Data Collection for the Parents and Staff Before and After the Intervention **eTable 1.** Number of Responses From the Staff per NICU and per Question **eTable 2.** Association Between Implementation Fidelity of NICUs or Other Factors and Total Family-Centered Care Ratings by Parents and Staff in the Linear Regression Model

Supplement 2.
Trial Protocol

Supplement 3.
Data Sharing Statement

## References

[1] Franck LS, O’Brien K. The evolution of family-centered care: from supporting parent-delivered interventions to a model of family integrated care. Birth Defects Res. 2019;111(15):1044-1059. doi:10.1002/bdr2.1521 31115181

[2] Gooding JS, Cooper LG, Blaine AI, Franck LS, Howse JL, Berns SD. Family support and family-centered care in the neonatal intensive care unit: origins, advances, impact. Semin Perinatol. 2011;35(1):20-28. doi:10.1053/j.semperi.2010.10.004 21255703

[3] Franck LS, Axelin A, Van Veenendaal NR, Bacchini F. Improving neonatal intensive care unit quality and safety with family-centered care. Clin Perinatol. 2023;50(2):449-472. doi:10.1016/j.clp.2023.01.007 37201991

[4] Darmstadt GL, Al Jaifi NH, Arif S, ; Care of Preterm or Low Birthweight Infants Group. New World Health Organization recommendations for care of preterm or low birth weight infants: health policy. EClinicalMedicine. 2023;63:102155. doi:10.1016/j.eclinm.2023.102155 37753445 PMC10518507

[5] Pallás Alonso C, Westrup B, Kuhn P, Daly M, Guerra P. Parental involvement. European Standards or Care for Newborn Health. Accessed February 8, 2024. https://newborn-health-standards.org/standards/standards-english/infant-family-centred-developmental-care/parental-involvement/

[6] National Institute for Health and Care Excellence. Babies, children and young people’s experience of healthcare. August 25, 2021. Accessed February 12, 2024. https://www.nice.org.uk/guidance/ng204

[7] Ding X, Zhu L, Zhang R, Wang L, Wang TT, Latour JM. Effects of family-centred care interventions on preterm infants and parents in neonatal intensive care units: a systematic review and meta-analysis of randomised controlled trials. Aust Crit Care. 2019;32(1):63-75. doi:10.1016/j.aucc.2018.10.007 30554939

[8] Segers E, Ockhuijsen H, Baarendse P, van Eerden I, van den Hoogen A. The impact of family centred care interventions in a neonatal or paediatric intensive care unit on parents’ satisfaction and length of stay: a systematic review. Intensive Crit Care Nurs. 2019;50:63-70. doi:10.1016/j.iccn.2018.08.008 30249426

[9] Oude Maatman SM, Bohlin K, Lilliesköld S, . Factors influencing implementation of family-centered care in a neonatal intensive care unit. Front Pediatr. 2020;8:222. doi:10.3389/fped.2020.00222 32435628 PMC7219204

[10] Mirlashari J, Brown H, Fomani FK, de Salaberry J, Zadeh TK, Khoshkhou F. The challenges of implementing family-centered care in NICU from the perspectives of physicians and nurses. J Pediatr Nurs. 2020;50:e91-e98. doi:10.1016/j.pedn.2019.06.013 31300252

[11] Carroll C, Patterson M, Wood S, Booth A, Rick J, Balain S. A conceptual framework for implementation fidelity. Implement Sci. 2007;2(1):40. doi:10.1186/1748-5908-2-40 18053122 PMC2213686

[12] Toivonen M, Lehtonen L, Löyttyniemi E, Ahlqvist-Björkroth S, Axelin A. Close Collaboration with Parents intervention improves family-centered care in different neonatal unit contexts: a pre-post study. Pediatr Res. 2020;88(3):421-428. doi:10.1038/s41390-020-0934-2 32380505 PMC7478938

[13] Toivonen M, Lehtonen L, Ahlqvist-Björkroth S, Axelin A. Effects of the Close Collaboration With Parents Intervention on the Quality of Family-Centered Care in NICUs. Adv Neonatal Care. 2023;23(3):281-289. 34596090 10.1097/ANC.0000000000000953

[14] Axelin A, Ahlqvist-Björkroth S, Kauppila W, Boukydis Z, Lehtonen L. Nurses’ perspectives on the close collaboration with parents training program in the NICU. MCN Am J Matern Child Nurs. 2014;39(4):260-268. doi:10.1097/NMC.0000000000000061 24978006

[15] Ahlqvist-Björkroth S, Axelin A, Korja R, Lehtonen L. An educational intervention for NICU staff decreased maternal postpartum depression. Pediatr Res. 2019;85(7):982-986. doi:10.1038/s41390-019-0306-y 30700835 PMC6760552

[16] Ahlqvist-Björkroth S, Axelin A, Setänen S, . Fewer maternal depression symptoms after the Close Collaboration with Parents intervention: two-year follow-up. Acta Paediatr. 2022;111(6):1160-1166. doi:10.1111/apa.16303 35181919 PMC9305419

[17] Des Jarlais DC, Lyles C, Crepaz N; TREND Group. Improving the reporting quality of nonrandomized evaluations of behavioral and public health interventions: the TREND statement. Am J Public Health. 2004;94(3):361-366. doi:10.2105/AJPH.94.3.361 14998794 PMC1448256

[18] Ahlqvist-Björkroth S, Boukydis Z, Axelin AM, Lehtonen L. Close Collaboration with Parents intervention to improve parents’ psychological well-being and child development: description of the intervention and study protocol. Behav Brain Res. 2017;325(Pt B):303-310. doi:10.1016/j.bbr.2016.10.020 27743940

[19] He FB, Axelin A, Ahlqvist-Björkroth S, Raiskila S, Löyttyniemi E, Lehtonen L. Effectiveness of the Close Collaboration with Parents intervention on parent-infant closeness in NICU. BMC Pediatr. 2021;21(1):28. doi:10.1186/s12887-020-02474-2 33430816 PMC7798198

[20] Ahlqvist-Björkroth S, Axelin A, Lehtonen L. Close Collaboration with Parents—implementation and effectiveness. Acta Paediatr. Published online March 21, 2024. doi:10.1111/apa.17210 38514910 PMC11894782

[21] Proctor E, Silmere H, Raghavan R, . Outcomes for implementation research: conceptual distinctions, measurement challenges, and research agenda. Adm Policy Ment Health. 2011;38(2):65-76. doi:10.1007/s10488-010-0319-7 20957426 PMC3068522

[22] Axelin A, Raiskila S, Lehtonen L. The development of data collection tools to measure parent-infant closeness and family-centered care in NICUs. Worldviews Evid Based Nurs. 2020;17(6):448-456. doi:10.1111/wvn.12475 33210818 PMC7756210

[23] Kitani M, Murakami H. The limiting distribution of combining the t and Wilcoxon rank sum tests. Statistics (Ber). 2020;54(4):871-884. doi:10.1080/02331888.2020.1809662

[24] Macdonald P. Power, type I, and type III error rates of parametric and nonparametric statistical tests. J Exp Educ. 1999;67(4):367-379. doi:10.1080/00220979909598489

[25] Wickham H, Averick M, Bryan J, . Welcome to the Tidyverse. J Open Source Softw. 2019;4(43):1686. doi:10.21105/joss.01686

[26] Dag O, Ilk O. An algorithm for estimating Box–Cox transformation parameter in ANOVA. Commun Stat Simul Comput. 2017;46(8):6424-6435. doi:10.1080/03610918.2016.1204458

[27] Bates D, Mächler M, Bolker BM, Walker SC. Fitting linear mixed-effects models using lme4. J Stat Softw. 2015;67(1):1-48. doi:10.18637/jss.v067.i01

[28] Ggplot2: elegant graphics for data analysis. Accessed February 27, 2023. https://ggplot2.tidyverse.org

[29] Wreesmann WW, Lorié ES, van Veenendaal NR, van Kempen AAMW, Ket JCF, Labrie NHM. The functions of adequate communication in the neonatal care unit: a systematic review and meta-synthesis of qualitative research. Patient Educ Couns. 2021;104(7):1505-1517. doi:10.1016/j.pec.2020.11.029 33341329

[30] Raiskila S, Lehtonen L, Tandberg BS, ; Separation and Closeness Experiences in Neonatal Environment (SCENE) research group. Parent and nurse perceptions on the quality of family-centred care in 11 European NICUs. Aust Crit Care. 2016;29(4):201-209. doi:10.1016/j.aucc.2016.09.003 27720034

[31] Labrie NHM, van Veenendaal NR, Ludolph RA, Ket JCF, van der Schoor SRD, van Kempen AAMW. Effects of parent-provider communication during infant hospitalization in the NICU on parents: a systematic review with meta-synthesis and narrative synthesis. Patient Educ Couns. 2021;104(7):1526-1552. doi:10.1016/j.pec.2021.04.023 33994019

[32] Benzies KM, Shah V, Aziz K, Lodha A, Misfeldt R. The health care system is making ‘too much noise’ to provide family-centred care in neonatal intensive care units: perspectives of health care providers and hospital administrators. Intensive Crit Care Nurs. 2019;50:44-53. doi:10.1016/j.iccn.2018.05.001 29759848

[33] Moreno-Sanz B, Alferink MT, O’Brien K, Franck LS. Family integrated care: State of art and future perspectives. Acta Paediatr. Published online May 13, 2024. doi:10.1111/apa.17272 38738866

[34] Benzies KM, Aziz K, Shah V, ; Alberta FICare Level II NICU Study Team. Effectiveness of Alberta Family Integrated Care on infant length of stay in level II neonatal intensive care units: a cluster randomized controlled trial. BMC Pediatr. 2020;20(1):535. doi:10.1186/s12887-020-02438-6 33246430 PMC7697372

[35] Prochaska JO, Velicer WF. The transtheoretical model of health behavior change. Am J Health Promot. 1997;12(1):38-48. doi:10.4278/0890-1171-12.1.38 10170434

[36] van Veenendaal NR, van Kempen AAMW, Franck LS, . Hospitalising preterm infants in single family rooms versus open bay units: a systematic review and meta-analysis of impact on parents. EClinicalMedicine. 2020;23:100388. doi:10.1016/j.eclinm.2020.100388 32548575 PMC7284081

[37] Kainiemi E, Flacking R, Lehtonen L, Pasanen M, Axelin A. Psychometric properties of an instrument to measure the quality of family-centered care in NICUs. J Obstet Gynecol Neonatal Nurs. 2022;51(4):461-472. doi:10.1016/j.jogn.2022.04.004 35598704

[38] Axelin A, Feeley N, Cambell-Yeo M, . Symptoms of depression in parents after discharge from NICU associated with family-centred care. J Adv Nurs. 2022;78(6):1676-1687. 34897769 10.1111/jan.15128 PMC9299776

[39] Itoshima R, Tuura K, Toome L, . Depressive symptoms in mothers of preterm infants before and during COVID-19 restrictions in neonatal intensive care units. Acta Paediatr. 2023;112(10):2164-2171. doi:10.1111/apa.16886 37354112

