# Supplementary material for: Outcomes Following Close Collaboration With Parents Intervention in Neonatal Intensive Care Units: A Nonrandomized Clinical Trial
Source: JAMA Netw Open. 2025 Jan 9;8(1):e2454099. doi: 10.1001/jamanetworkopen.2024.54099 (PMC11718553; doi:10.1001/jamanetworkopen.2024.54099)
Supplement: Supplement 1. — eFigure. Periods of Intervention and Data Collection for the Parents and Staff Before and After the Intervention eTable 1. Number of Responses From the Staff per NICU and per Question eTable 2. Association Between Implementation Fidelity of NICUs or Other Factors and Total Family-Centered Care Ratings by Parents and Staff in the Linear Regression Model [file jamanetwopen-e2454099-s001.pdf]

Supplemental Online Content

Itoshima R, Varendi H, Toome L, et al. Outcomes following Close Collaboration With Parents intervention in neonatal intensive care units: a nonrandomized clinical trial. *JAMA Netw Open.* 2025;8(1):e2454099. doi:10.1001/jamanetworkopen.2024.54099

eFigure. Periods of Intervention and Data Collection for the Parents and Staff Before and After the Intervention

eTable 1. Number of Responses From the Staff per NICU and per Question

eTable 2. Association Between Implementation Fidelity of NICUs or Other Factors and Total Family-Centered Care Ratings by Parents and Staff in the Linear Regression Model

This supplemental material has been provided by the authors to give readers additional information about their work.

© 2024 Itoshima R et al. *JAMA Network Open*.

eFigure. Periods of Intervention and Data Collection for the Parents and Staff Before and After the Intervention.

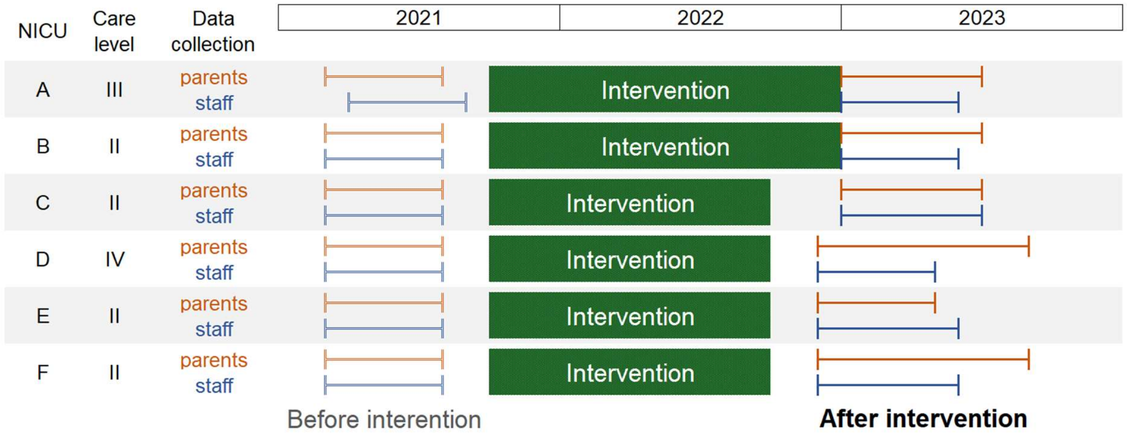

The intervention period in each NICU is shown as a green square. The data collection periods in each NICU before and after the intervention are illustrated for the parents (orange) and staff (blue). Care level of each NICU is also summarized.

NICU, neonatal intensive care unit.

© 2024 Itoshima R et al. *JAMA Network Open*.

eTable 1. Number of Responses From the Staff per NICU and per Question

n (%)	Before (n=7,448)	After (n=6,717)
**NICU**		
A	500 (6.7)	238 (3.5)
B	2762 (37.1)	2611 (38.9)
C	770 (10.3)	910 (13.5)
D	1032 (13.9)	588 (8.8)
E	976 (13.1)	1164 (17.3)
F	1408 (18.9)	1206 (18.0)
**Question**		
Q1 Active listening	849 (11.4)	743 (11.1)
Q2 Participation in care	843 (11.3)	741 (11.0)
Q3 Individualized guidance	828 (11.1)	746 (11.1)
Q4 Shared decision making	833 (11.2)	741 (11.0)
Q5 Mutual trust (parents→staff)	822 (11.0)	732 (10.9)
Q6 Mutual trust (staff→parents)	829 (11.1)	723 (10.8)
Q7 Individualized information	809 (10.9)	748 (11.1)
Q8 Emotional support	843 (11.3)	739 (11.0)
Q9 Participation in medical rounds	792 (10.6)	804 (12.0)

The number of responses in each NICU and for each question is summarized. The proportion of responses is also calculated.

NICU, neonatal intensive care unit.

© 2024 Itoshima R et al. *JAMA Network Open*.

eTable 2. Association Between Implementation Fidelity of NICUs or Other Factors and Total Family-Centered Care Ratings by Parents and Staff in the Linear Regression Model

	Parents [a]		Staff [a]	
	β (95% CI)	*P*	*β* (95% CI)	*P*
After-intervention	**5.9 (0.4, 11.0)**	**0.04**	**2.3 (1.4, 3.2)**	**<0.001**
High fidelity NICU	1.5 (-4.0, 7.1)	0.59	**1.7 (0.8, 2.7)**	**<0.001**
After-intervention * High fidelity NICU [b]	2.7 (-4.4, 9.8)	0.46	**2.1 (0.8, 3.4)**	**0.002**
Level II NICU	**26.0 (20.0, 32.0)**	**<0.001**	**8.6 (9.6, 7.6)**	**<0.001**
Birth weight	0.00 (0.00, 0.00)	0.67	NA	NA
Estonian/Russian as native language	-5.2 (-15.0, 4.4)	0.29	NA	NA

The linear regression model for the ratings by the parents included the following factors: intervention (after or before), fidelity of NICU (high or low), care level of NICU (level II or III/IV), birth weight as a continuous variable, mother tongue (Estonian/Russian or others) and interaction between intervention and fidelity. The linear regression model for the ratings by the staff included the following factors: intervention (after or before), fidelity of NICU (high or low), care level of NICU (level II or III/IV), and interaction between intervention and fidelity.

[a] Box-cox transformation was adopted for the dependent variables and β was calculated with the transformed variables.

[b] Interaction between intervention (before-after) and fidelity (high-low).

95% CI, 95% confidence interval; NICU, neonatal intensive care unit.

© 2024 Itoshima R et al. *JAMA Network Open*.
