# Supplementary material for: Outcomes Following Close Collaboration With Parents Intervention in Neonatal Intensive Care Units: A Nonrandomized Clinical Trial
Source: JAMA Netw Open. 2025 Jan 9;8(1):e2454099. doi: 10.1001/jamanetworkopen.2024.54099 (PMC11718553; doi:10.1001/jamanetworkopen.2024.54099)
Supplement: Supplement 2. — Trial Protocol [file jamanetwopen-e2454099-s002.pdf]

Research protocol– Sari Ahlqvist-Björkroth

The impact of the Close Collaboration with Parents intervention – Estonian study

Principal investigator: Sari Ahlqvist-Björkroth, Doctor of psychology, postdoctoral researcher
Affiliation: Clinical medicine, Discipline of pediatrics, University of Turku.
Address: University of Turku, 20014.
Gms: +358405119600
Email address: sarahl@utu.fi

Research team:
Liisa Lehtonen, Professor in Pediatrics, University of Turku, and Director of the Division of Neonatology, Turku University Hospital
Anna Axelin, Associate Professor in Nursing Science, University of Turku
Liis Toome, MD, PhD, Head of the Department of Neonatal and Infant Medicine, Tallinn Children's Hospital
Mari-Liis Ilmoja, MD, senior pediatrician, director of the PNICU, Tallinn Children's Hospital
Pille Saik, MD, senior pediatrician, head of the Neonatal Unit, West-Tallinn Central Hospital
Heili Varendi, MD, PhD, senior pediatrician, head of the NICU at Tartu University Hospital and Associate Professor in Pediatrics, University of Tartu
Tuuli Metsvaht, MD, PhD, head of the PNICU at Tartu University Hospital and Professor in Neonatal and Child Intensive Care, University of Tartu
Ryo Itoshima MD, PhD student, University of Turku

Over 10% of all newborn infants require hospital care shortly after birth, many due to prematurity.[1] Hospitalization for a premature infant can last from a few weeks to several months. A recent study showed that preterm infants were left in isolation, without any human contact, during about 80% of their hospital stays.[2] Parents' role and participation in their infant's care are severely limited by the unit policies in most neonatal care settings. Another recent study, conducted in 11 neonatal intensive care units (NICUs), showed that the mean time either parent was present varied from 3.3 to 22.3 hours, and the mean time for skin-to-skin contact varied from 0.3 to 6.6 hours per day.[3] Preterm infants are at risk of later developmental problems, especially regarding behavioral outcomes. These problems are partly caused by early parent–infant separation and a lack of parents' participation in the care of their infant.[4]

Early separation from the premature infant also has negative impacts on parents and their parenting. Parents of preterm infants have reported feelings of separation when their infant is admitted to an NICU. Separation makes parents feel insufficient, unnecessary, and as if they are not part of the infant's care team.[5] A prolonged early separation may also lead to parental depression.[6] Specifically, parents of prematurely born infants are at higher risk of postpartum depression (PPD), with rates varying from 6 to 48 percent. The depression scores of some parents remain high (17–20%) until the second year after the preterm delivery.[7,8] Depression has typically a strong comorbidity with anxiety. Although, anxiety is a separate mood disorder having a different symptomatology than depression. NICU parents have also an elevated risk for anxiety symptoms with rates varying from 18 to 72 percent.[9,10,11]

The problems mentioned above are well recognized by neonatal professionals, and thus family-centered care (FCC), is strongly advocated and considered as “state of the art” in modern neonatal intensive care. FCC has been shown to improve neonatal, parental, and health service outcomes.[4] However, there is no consensus regarding FCC practices, leading to confusion in its implementation. Developmental psychology theories (DPT) are grounded on strong empirical research and are better operationalized than FCC, and thus they can guide the content of interventions and implementation as well as the choice of outcomes when evaluating the effectiveness of FCC.[12]

We have developed an educational intervention that is based on knowledge from DPTs, the nursing sciences, and evidence-based medicine.[13] The intervention, called “Close Collaboration with Parents” is targeted at hospital nursing and medical staff to improve their skills to support the early parent–infant relationship. By educating the whole staff of a unit, we can make the benefits available for all patient care in the unit. The innovation of the intervention is related to its multiprofessional approach and the use of experiential learning with a mentor as a facilitation method.[14] We have implemented and studied the intervention in 12 hospitals in Finland between the years 2009 and 2020. Our research shows that the Close Collaboration with Parents training improves units’ FCC practices,[15] increases parents’ presence during hospital care by 37%, increases parents’ skin-to-skin care by 50%,[16] and decreases the percentage of mothers who scored over the probable clinical depression cutoff from 10% to 2%.[17] Staff have reported that the training helped them to change their attitudes and trust parents as caretakers.[18]

Close Collaboration with Parents is a complex intervention because it aims to improve the care model of the unit. To fully understand the functioning of a complex intervention, a systematic approach is recommended for conducting process evaluations and identifying key impact mechanisms.[19] However, there are no previous studies about the impact mechanisms of FCC interventions to explain how the interventions produce positive changes in parental outcomes.

Research Objectives and Methods

The overall goal is to address the knowledge gap related to the impact mechanisms of FCC interventions on improvements in patient outcomes. The specific goal is to test a potential impact mechanisms model of the Close Collaboration with Parents intervention (Figure 1).

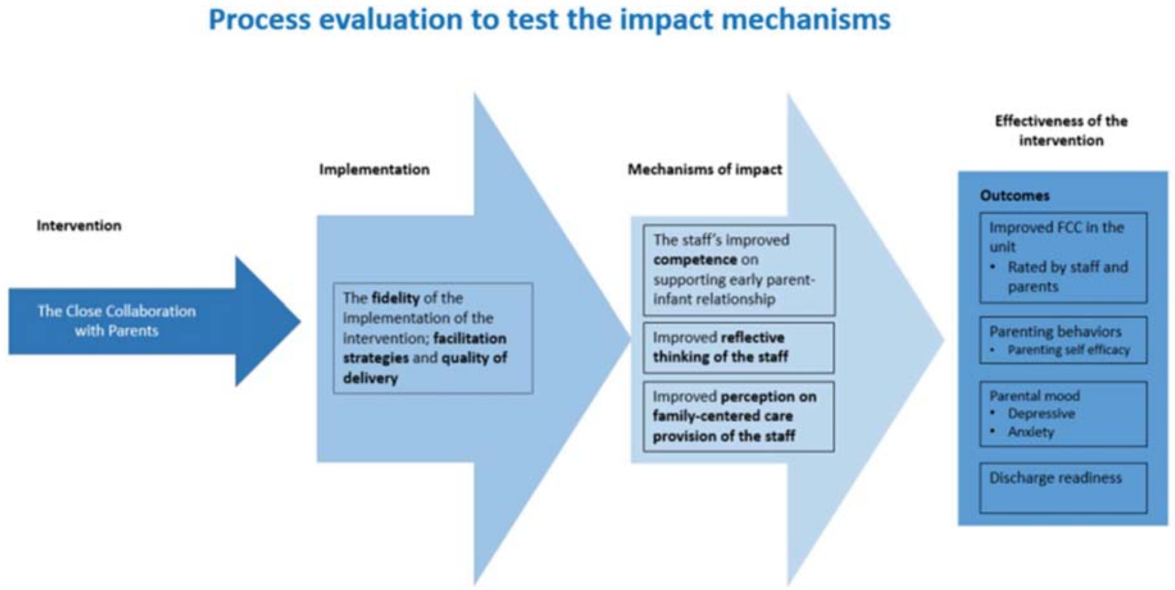

Figure 1. Process evaluation is used as a theoretical framework to construct a logical model to explain the change mechanisms of Close Collaboration with Parents.

The specific research questions are as follows:

1. What is the achieved level of fidelity in the implementation process of the intervention in the Estonian context?
2. Does the intervention promote immediate changes in the staff's collaboration competence, perception of the FCC care provision, and reflective thinking?
3. Does the intervention have a significant positive effect on the FCC practices of the units?
4. Does the intervention have a significant positive effect on parenting self-efficacy, parental mood and parents' readiness for discharge at the time of hospital discharge?
5. If the answers to questions 2, 3, and 4 are positive, we will then seek to answer whether the potential impact mechanisms mediate the improvements in unit FCC practices, parental self-efficacy, mood and readiness for discharge.

Materials and Methods

<u>Design</u>

A quasi-experimental before and after intervention design is used to test the effect of the intervention, where the impact mechanisms are treated as mediators of the change.

<u>Sample</u>

The subjects are employees of five neonatal units of three Estonian hospitals participating in the Close Collaboration with Parents intervention (hereinafter: staff) and newborns cared for in the units and their parents (hereinafter: families). Three of the units are at Tallinn: West-Tallinn Maternity Hospital (NICU and neonatal unit), the Department of Anesthesiology and Intensive Care (PICU/level IIIB) and the Department of Neonatal and Infant Medicine (NICU/level II) at the Children's Hospital. The Department of Anesthesiology and Intensive Care is an open-bay intensive care unit, while the Department of Neonatal and Infant Medicine is a single-family-room unit. Another two units are located in Tartu University Hospital: the Department of Neonatal and Pediatric Intensive Care (PNICU, level IIIC) and a stepdown unit (level II). PNICU is an open-bay unit while the stepdown unit has two open-bay intensive care rooms (mothers have their bed in another room of the ward) and 8 single-family rooms.

<u>The inclusion criteria for the families are</u>: 1) families whose newborns have been hospitalized during the first 28 days of infant life, 2) expected length of stay of the newborn is at least three days, 3) discharge is planned to happen within a week, and 4) at least one parent has agreed to participate in the study. <u>The staff working in the units during the entire study period is eligible for the study.</u>

Families are recruited during a four-month period before and after the intervention, with the aim to recruit at least 50 families per participating hospital in both periods. Some newborns may be treated in a different hospital or in different units of the same hospital. In the case of a transfer, the family is asked to fill in only the Family Center questionnaire (Appendix 10) if the newborn has been in that unit for more than 72 hours.

<u>Data collection</u>

The pre-intervention data is collected in spring 2021 during a four-month period when the training on unit mentors is going on, but the implementation to the unit has not begun. The data measuring the fidelity of intervention implementation will be collected between January 2021 and October 2022. The post-intervention data is collected in the shift of the years 2022 and 23 during a four-month period. (Figure 2).

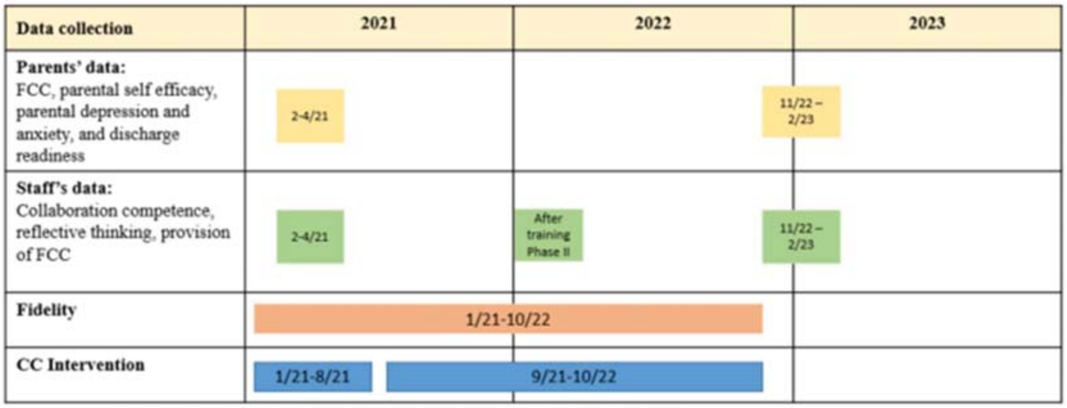

Figure 2. Illustrates the timeline of the data collection.

A log will be kept of all infants discharged to home from the unit i.e. to evaluate drop-out rate and biases (Appendix A). The log includes the patient's gestational age, birth weight, diagnosis, type of feeding at discharge, and the length of hospital stay. If there is missing information in the log, it is completed with the information from the medical records. A log is also kept to follow the units' restrictions related to Covid-19 and concerning family visitation and participation policies (Appendix B/C).

Methods

Methods to assess the fidelity

The fidelity is analysed by tracking the participants' behaviour in the e-learning module by analytic softwares, such as Google Analytics, Hotjar and Vimeo. The participants are informed about this and they are asked for their written consent and agreement when entering the module for the first time. Furthermore, a log is kept about the practices the mentors are carrying out with their colleagues in the units (Appendix D).

Methods to assess the impact of the intervention on staff

Background questionnaire
A questionnaire is used to collect background information of staff members (Appendix 1).

Collaboration competence questionnaire
A questionnaire is used to assess the collaboration competence of the staff at the beginning of the intervention, in the middle of the implementation and in the end. The questionnaire is specifically created to assess competencies that are relevant for the Close Collaboration with Parents training program. The questionnaire includes 21 questions that are evaluated with scale from 1 to 10 (1=not competent at all, 10=extremely competent). If the question does not ably the daily work role of the professional option "Doesn't belong to my role" can be used. (Appendix 2)

The reflective thinking
The level of reflective thinking of the staff is measured using the Reflective Thinking Measure[20] (Appendix 3). The 16-question measure determines whether an individual engages in reflective thinking and to what extent s/he does that. The questions are answered with options varying from strongly agree, which is scored as 5, through to strongly disagree, which is scored as 1. Scores range from 16 to 80, with 80 indicating the highest possible level of reflection. The 16 questions form four factors: 1) Habitual Actions, 2) Understanding, 3) Reflection and 4) Critical reflection. The structural validity of the scale has been tested with health care professionals and it has shown to be good.[20]

The level of reflective thinking is also assessed with questions related to a fictitious case (Appendix 4). The case represents so called 'critical incident' that the staff is asked to describe from three perspectives: (a) how they would normally act in a similar situation in their unit; (b) a general meaning and reflect how

the general meaning resonates with their own experience; and (c) potential new ways of acting in the situation that would provide better support for the family.[21]

The FCC care provision

The perception of multiprofessional staff members on parent support they have given that day is measured with DigiFCC web-questions[22]. The question will reflect the staff's point of view on the support provided for parents in following dimensions: 1) active listening, 2) parent participation in infant care, 3) individualized parent education, 4) parent participation in decision-making, 5) the parent trust staff in infant care, 6) the staff feel the parents trust them in infant care, 7) participation in medical round/medical care decision making, 8) information sharing, and 9) emotional support. Staff members answer the questions through a website which is always open at the research computer in the unit. The computer is connected to the Internet. Every staff member (regardless of qualifications) working in the participating units is asked to answer a daily question through a website after their each work shift for a 3-month period. There are 9 questions which are randomized in 3-question blocks. Answers are numbers from 1 to 5 or 0 if the staff member hasn't been working with the parents on that shift or didn't participate in the medical round. In addition to the number, they had space to write free text. The staff members are provided with the instructions of Web-questions.

Regarding the same 3-month period, the researcher/administrator collects the number of nursing staff work shifts in the unit to calculate the response rate. The research will create a questionnaire for each unit through the Master account website. The responsible researcher will have an access to Research account to collect the answers.

Methods to assess the impact of the intervention on parents

Infant-parent background questionnaire
A questionnaire is used to collect background information of infants/parents (Appendix 5)

Parents' depressive symptoms
The depressive symptoms of parents are measured with Edinburgh Postnatal Depression Scale (Appendix 6) (EPDS).[23] The 10-question EPDS is a valid method to measure depressive symptoms both in mothers and in fathers. The 10-question scale includes symptoms such as insomnia, mood, tearfulness, and the thoughts of self-harm. The scale asks how the parent has felt during the previous week. The parents rate each question on a scale from 0 to 3. The maximum score on the EPDS would be 30. Parents are asked to fill in the EPDS questionnaire from two to one week before discharge. The EPDS is easy to administer and has high sensitivity as a screening tool.

Parent's anxiety symptoms
The anxiety symptoms of the parents are assessed with State and Trait Anxiety Inventory (STAI)[24] (Appendix 7). State anxiety is an emotional reaction that varies from one situation to another, whereas the trait anxiety is a personality characteristic. The STAI measurement provides separate scores for both types of anxiety. There are 20 questions about state anxiety that are rated with 4-point scale (1=not at all,

4=very much) and 20 questions about trait anxiety that are also rated with 4-point scale (1=almost never, 4=almost always). The recommended cut-off for the postnatal anxiety measured with STAI-S is 34/80.[25]

The research assistant will calculate the parent's scores of EPDS and STAI-S when the questionnaires are returned. She informs the responsible researcher of the unit if the total score of the EPDS is >12 or if the parent has suicidal thoughts and if the total score of STAI-S is >33. The local research group will then decide who will contact the parent and refer her to appropriate health care services if needed.

Parenting self-efficacy scale

Parenting self-efficacy is measured Perceived Maternal Parenting Self-Efficacy (PMP S-E) instrument[26] (Appendix 8). The PMP S-E instrument consists of 20 items (scoring ranges 20–80) with four subscales: 1) care taking procedures, 2) evoking behaviors, 3) reading behaviors or signaling, and 4) situational beliefs. The items are rated with a four point Likert scale ranging from 'strongly disagree' (score 1) to 'strongly agree' (score 4). A low score on this scale indicates a low maternal self-efficacy. The PMP S-E tool is a psychometrically robust, reliable and valid measure of parenting self-efficacy in mothers of relatively healthy hospitalized preterm neonates.[24]

Parent's readiness for discharge

The readiness for the discharge is measured with the Readiness for Hospital Discharge Scale (RHDS)[27] (Appendix 9). The questionnaire includes 23 items. Four attributes of readiness for discharge are measured within the subscales: Personal Status (7 items), Knowledge (7 items), Coping Ability (4 items), and Expected Support (5 items). Answers are on a 10-point scale from 0 to 10 (e.g., not at all, totally). The RHDS has shown to be a reliable and valid measure of patients' perception of readiness for discharge.

Parent's perceived FCC

The parent participation and the support parents feel they receive from the staff is measured using questionnaire modified from DigFCC SMS questions (Appendix 10).[22] The questionnaire includes 9 questions covering following aspects of staff support 1) active listening, 2) parent participation in infant care, 3) individualized parent education, 4) parent participation in decision-making, 5) the parent trust staff in infant care, 6) the parent feel the staff trust them in infant care, 7) participation in medical round/medical care decision making, 8) received information, and 9) emotional support. The parents are asked to rate separately their experience of the each hospital unit they have stayed during the hospitalization of their baby. They are asked to answer the question with the Likert scale from 1 to 7 (1=not at all–7=very much; 0= if they did not visit the unit). This questionnaire can be handed out solely for the families that have stayed in a unit at least three days but their infant is transferred to another unit.

<u>Data management</u>

We will utilize REDcap (Research Electronic Data capture) for the data transfers from Estonia to Finland and for the data storage. REDcap is an electronic tool for data collection that is provided from the server facilities owned and operated by the University of Turku IT Services, which also means all data collected

to the system is stored and kept at the University of Turku. When the data is stored to the online file storage of Turku University it is automatically copied to three independent servers that also function as data backup systems. The data is entered to the REDcap system by the local research assistant/ research nurse.

Data security description:
- Centralized administration by separate administration accounts and compartmentalized user privileges
- Use of current and reliable hardware, and software which is updated continuously
- Centralized identity management (IDM)
- Locally administered industry-standard network infrastructure and services
- Common server platforms with minimal tailoring
- Continuous monitoring of network traffic patterns and service statistics
- Widely deployed security software and other monitoring tools

The PI controls the access to the data. The co-researchers can approach the PI to request access to the data. The PI will apply to the University of Turku IT Services to provide the access to the online storage. If a co-researcher is not working at the University of Turku, the PI can ask the IT services to give that person a visitor status and, thereby, an access to the online file storage. All new users are identified using government-issued identification documents or by citizens' identification and payment service. The allowance to access the data is issued only for the active research period and ends when the research is completed. The IP will keep a record of those who have active access to the data.

All the data will be handled anonymously. The participants will get an ID code that is used when data is stored and analyzed. The recruitment log of the parents and staff members are used to undo the ID codes and, therefore, they are kept separately from the data files. The analytic softwares (Google Analytics, Hotjar and Vimeo) also track anonymously participants' behavior on the e-learning module's website. The personal details (password, email, etc) are not recorded and the IP addresses are anonymized by cutting out the last few digits, so only the rough geographical area of access to the e-learning module is identified. The logs and all the data will be destroyed five years after the last publication from the data.

<u>The intervention</u>

The Close Collaboration with Parents intervention will be carried out in the four NICUs in Estonia. The delivery of the intervention follows a structure that has been developed and fine-tuned between 2009 and 2020 when it was implemented in 14 NICUs. First, experienced nurses or doctors from the units will undergo a three-week training to become "unit mentors," and then these unit mentors will train other members of staff. The training of the units is strongly based on experiential learning utilizing bedside practices, reflection of the practices, and application of this knowledge in everyday clinical situations. Theoretical teaching, which precedes clinical bedside mentoring, will occur through a new e-learning tool. Each staff member will spend about four working days training alongside his or her routine work. The unit mentors will function as facilitators for all the staff, and they have dedicated time for this. The content of the bedside practices is guided by a manual. To support the delivery of this phase, the training

team will provide frequent remote supervision for the unit mentors and leadership. The units have committed to allocate the resources for the implementation of the intervention. The applicant will have the main responsibility for the delivery of the training. The aim is to train about 75% of the staff members in the units during the 18-month period.

Translation process

The following study material needs to be translated by official translator/linguistic to Estonian and Russian and by an independent other official translator/linguistic back to English: the Collaborative Competence -questionnaire, the Reflective Thinking Measure, and Perceived Maternal Parenting Self-Efficacy Instrument, Readiness for Hospital Discharge Scale. After the back-translation, a cognitive debriefing session will be organized to check the validity of the translation. Other methods already exist in Estonian and Russian. Also the following study material can be translated by a translator who is fluent in English and target language: information leaflets about study, informed consent forms and the instructions for the participants.

Ethical considerations

An ethical approval will be asked for the study protocol according to the regulations of each participating hospital. The study protocol needs to get a favourable statement from the Ethics Committee in each country before the data collection can begin. Written informed consent will be obtained from each staff member and parent before study participation. The analytic software (Google Analytics, Hotjar and Vimeo) will be activated only after the consent has been obtained from a participant. The application for ethics should include an opportunity to complete the information received from the parents (infant and family characteristics questionnaire) using patient medical records.

Conducting the Research: The tasks of the Local Research Assistant/ Research Nurse

1. **Initiation of the study (1-2 days)**

- To inform the unit and set up the study
- to make information leaflets available, computer setup, reinforcement

2. **Recruitment by a research assistant or staff members (nurses/head nurses/doctors) responsible 1-1½ hours per every weekday**

- to check daily the patients who are going to be discharged within a week
- to approach the families with the written study information
- to approach the families for the written consent the following day
- to collect the information of the non-recruited patients (see Appendix A_Log for Study)
- to handout the questionnaires to the recruited families and collect the filled in questionnaires before the baby is discharged. Resources are needed for recruitment for a 3-month period before the intervention and 3-month period after the intervention. The work time is estimated to be from ½ to 1 hour on each week day (preferably also on weekends).

3. **Data entry to the REDcap**
    - not included in this time allocation

Finances

The units are responsible for the following costs related to the study. At least following costs need to be considered**:**

- Intervention costs (training fee + salary of the mentor nurses)
- Material (printing out the informed consent forms, information and instruction for the parents and staff, diaries)
- Desk computer/laptop and Internet access available for the study period for the web questionnaire
- Cost of the ethical approval
- Cost of research staff/ nursing staff participation
- Data entry

Data analysis is centralized and paid in Finland.

Authorship and ownership of the data

NEJM 1997 criteria for authorship. Authors to include are the research team members and maximum one additional author per a collaborating center. The data is co-owned by the research team and the use of the data and its publication plan must be separately negotiated for each paper within the research team.

Implications of the study

The results of this study should be transferable to other neonatal units. The identified significant impact mechanisms can guide quality improvements in neonatal care globally. When the change can be made at the unit level, it will benefit all babies—and their parents—cared for in the hospitals. Improving the mental health of NICU parents would also extend the benefits to the whole of society.

Bibliography

1. The European Perinatal Health Report. http://www.europeristat.com/reports/european-perinatal-health-report-2010.html.
2. Gonya J, Feldman K, Brown K, Stein M, Keim S, Boone K, Rumpf W, Ray W, Chawla N, Butter E. Human interaction in the NICU and its associations with outcomes on the Brief Infant-Toddler Social and Emotional Assessment (BITSEA). Early Hum Dev 2018;127: 6-14.
3. Raiskila S, Axelin A, Toome L, Caballero S, Montirosso R, Normann E, Hallberg B, Ewald U, Lehtonen L. Parents' presence and parent–infant closeness in 11 NICUs in six European countries varies between and within countries. Acta Paediatr 2017;106(6):878-888.
4. Flacking R, Lehtonen L, Thomson G, Axelin A, Ahlqvist S, Moran VH, et al. Closeness and separation in neonatal intensive care. Acta Paediatr. 2012;101(10):1032–7.
5. Wigert H, Johansson R, Berg M, Hellstrom A. Mothers' experiences of having their newborn child in a neonatal intensive care unit, Scand. J. Caring Sci. 2006;20 (1): 35–41.

6. Feldman R, A. Weller, J.F. Leckman, J. Kuint, A.I. Eidelman, The nature of the mother's tie to her infant: maternal bonding under conditions of proximity, separation, and potential loss, J. Child Psychol. Psychiatry. 1999;40: 929–939.
7. Pace CC. et al. Evolution of depression and anxiety symptoms in parents of very preterm infants during the newborn period. JAMA Pediatr. 2016;170: 863–870.
8. Vigod, S., Villegas, L., Dennis, C. & Ross, L. Prevalence and risk factors for post- partum depression among women with preterm and low-birth-weight infants: a systematic review. BJOG: Int. J. Obstet. Gynaecol. 2010;117: 540–550.
9. Bonacquisti A, Geller PA, Patterson CA. Maternal depression, anxiety, stress, and maternal-infant attachment in the neonatal intensive care unit. Journal of Reproductive and Infant Psychology. 2020;38(3): 297-310. DOI: 10.1080/02646838.2019.1695041
10. González-Hernández A, González-Hernandez D, Fortuny-Falconi CM, Tovilla-Zárate CA, Fresan A, Nolasco-Rosales GA, … Escobar Chan YM. Prevalence and Associated Factors to Depression and Anxiety in Women with Premature Babies Hospitalized in a Neonatal Intensive-Care Unit in a Mexican Population. Journal of Pediatric Nursing. 2019;45: e53-e56.
11. Trumello C, Candelori C, Cofini M, Cimino S, Cerniglia L, Paciello M, Babore A. Mothers' Depression, Anxiety, and Mental Representations After Preterm Birth: A Study During the Infant's Hospitalization in a Neonatal Intensive Care Unit. Frontiers in Public Health. 2018;6:359.
12. Al-Motlaq M A, Carter B, Neill S, Hallstrom IK, Foster M, Coyne I, …Shields L. Toward developing consensus on family-centred care: An international descriptive study and discussion. Journal of Child Health Care. 2019;23(3): 458–467.
13. Ahlqvist-Björkroth S, Boukydis Z, Axelin A, Lehtonen L. Close Collaboration with Parents™ Intervention to improve parents' psychological well-being and child development. Description of the intervention and study protocol. Behavioural Brain Research 2017;325: 303–310.
14. Toivonen M, Lehtonen L, Ahlqvist-Björkroth S. et al. Key factors supporting implementation of a training program for neonatal family-centered care – a qualitative study. BMC Health Serv Res 2019:19; 394.
15. Toivonen M, Lehtonen L, Löyttyniemi E, Ahlqvist-Björkroth S, Axelin A. Close Collaboration with Parents intervention improves family centered care in different neonatal unit contexts – a pre-post study (Submitted: Pediatric Research)
16. He F, Axelin A, Ahlqvist-Björkroth S, Raiskila S, Löyttyniemi E, Lehtonen L. Parent-infant closeness increased by Close Collaboration with Parents intervention - before and after study in nine NICUs (Submitted)
17. Ahlqvist-Björkroth S, Axelin A, Korja R, Lehtonen L. An educational intervention for NICU staff decreased maternal postpartum depression. Pediatr Res. 2019;85(7):982-986.
18. Axelin A, Ahlqvist-Björkroth S, Kauppila W, Boukydis Z, Lehtonen L.Nurses' Perspective on Close Collaboration with Parents Training Program in the NICU. Am J Matern Child Nurs 2014, 39:260-68.
19. Moore G, Audrey S, Barker M, Bond L, …Baird J. Process evaluation of complex interventions: Medical Research Council guidance. BMJ 2015;350: 1258.

20. Kember D, Leung DYP, Jones A, et al. Development of a questionnaire to measure the level of reflective thinking. Assessment & Evaluation in Higher Education. 2000;25(4): 381-395.
21. Griffin ML. Using critical incidents to promote and assess reflective thinking in preservice teachers. Reflective Practice. 2003;4(2): 207-220.
22. Axelin A, Raiskila S, Lehtonen L. The Development of Data Collection Tools to Measure Parent–Infant Closeness and Family-Centered Care in NICUs. Worldviews on Evidence-Based Nursing. 2020;17: 448-456.
23. Cox JL, Holden JM, Sagovsky R. Detection of postnatal depression. Development of the 10-item Edinburgh Postnatal Depression Scale. BJ Psych 1989;150: 782-786.
24. Spielberge CD, Gorsuch, RL, Lushene R, Vagg PR, Jacobs GA. (1983). Manual for the State-Trait Anxiety Inventory. Palo Alto, CA: Consulting Psychologists Press.
25. Tendais I, Costa R, Conde A, and Figueiredo B. Screening for Depression and Anxiety Disorders from Pregnancy to Postpartum with the EPDS and STAI. Spanish Journal of Psychology. 2014;17; 1–9.
26. Barnes CR, Adamson-Macedo EN. Perceived Maternal Parenting Self-Efficacy (PMP S-E) tool: development and validation with mothers of hospitalized preterm neonates. Journal of Advanced Nursing. 2007;60(5): 550–560
27. Weiss ME, Piacentine LB. Psychometric Properties of the Readiness for Hospital Discharge Scale. Journal of Nursing Measurement. 2006;14(3): 164-180.
