# Supplementary material for: Outcomes Following Close Collaboration With Parents Intervention in Neonatal Intensive Care Units: A Nonrandomized Clinical Trial
Source: JAMA Netw Open. 2025 Jan 9;8(1):e2454099. doi: 10.1001/jamanetworkopen.2024.54099 (PMC11718553; doi:10.1001/jamanetworkopen.2024.54099)
Supplement: Supplement 3. — Data Sharing Statement [file jamanetwopen-e2454099-s003.pdf]

Data Sharing Statement

Itoshima. Outcomes Following Close Collaboration With Parents Intervention in Neonatal Intensive Care Units. *JAMA Netw Open*. Published January 09, 2025. doi:10.1001/jamanetworkopen.2024.54099

Data
Additional Information: ClinicalTrials.gov NCT06258655
Data available: No

Additional Information
Explanation for why data not available: The individual patient data are not be shared due to privacy considerations.
